# Beyond pleasure: exploring the role of sex toys in female sexual functioning and wellbeing—From enhancement to functional necessity

**DOI:** 10.3389/fpubh.2026.1771881

**Published:** 2026-04-23

**Authors:** Maciej Stokłosa, Iga Florczyk, Gniewko Wiȩckiewicz, Karolina Kiersten, Magdalena Piegza, Robert Pudlo

**Affiliations:** 1 Department and Clinic of Psychiatry, Faculty of Medical Sciences in Zabrze, Medical University of Silesia, Tarnowskie Góry, Poland; 2 Department of Gynecology, Obstetrics and Oncological Gynecology, Faculty of Medical Sciences in Zabrze, Medical University of Silesia, Bytom, Poland

**Keywords:** health promotion, orgasm quality, sex toys use, sexual risk behaviors, sexual wellbeing

## Abstract

**Background:**

Sex toy use is a common yet complex component of female sexuality, often serving dual roles as an enhancement tool or a functional necessity. Grounded in a biopsychosocial framework, this study examined the associations between sex toy usage patterns, sexual functioning, and sexual risk behaviors in young adult women.

**Methods:**

An online cross-sectional survey was conducted among 199 female university students using the Female Sexual Function Index (FSFI), the Sexual Risk Survey (SRS), and a study-specific questionnaire on erotic accessory usage.

**Results:**

Over 50% of women reported using sex toys during masturbation, and 30% integrated them into partnered activity. Multivariate regression analysis revealed that while the ‘perceived necessity of a sex toy for orgasm' was a significant independent negative predictor for specific FSFI domains- arousal, lubrication and orgasm -it did not independently predict the total FSFI score. Furthermore, frequent toy use during masturbation emerged as an independent positive predictor of FSFI arousal domain. Higher frequency of sex toy use across both solo and partnered contexts was independently associated with an increased propensity for sexually risky behaviors across all SRS domains.

**Conclusions:**

Incorporating erotic accessories serves as a positive intimacy-enhancing strategy in stable relationships, while subjective reliance on these devices represents a functional adaptation to reach physiological thresholds rather than a marker of global sexual impairment. The correlation between frequent usage and elevated sexual risk scores underscores the need for stigma-sensitive sexual health education focused on safe exploration and hygiene.

## 1 Introduction

The rapid expansion of the sex toy market in recent years has made these products an important component of many people's intimate lives. Studies from various countries show that their use is not a marginal but a widespread phenomenon: in Germany, more than half of adults report using sex toys in partnered sexual activity, and almost half during solo activity (1). Recent comparative analyses further suggest that owning and using sex toys is associated with greater sexual satisfaction and higher overall quality of life (2). These findings are consistent with clinical and observational data indicating that regular vibratory stimulation may improve sexual functioning (desire, arousal, lubrication, orgasm) and pelvic health–related quality of life, as well as alleviate vulvodynia-related pain. Consequently, sex toy use is increasingly supported in systematic reviews as an adjunctive intervention in sexology and urogynecology (3, 4).

Orgasm is one of the key domains of women's sexual functioning, and difficulties in reaching orgasm or reduced orgasmic intensity are among the most commonly reported sexual problems (5). Contemporary models emphasize the biopsychosocial nature of sexual dysfunctions, highlighting the interplay of biological, psychological and relational factors, as well as the role of individual coping strategies, such as modifying forms of stimulation or exploring one's own sexuality (6). This study is grounded in a biopsychosocial framework of human sexuality, viewing the use of erotic accessories not as inherently pathological, but as a normative component of contemporary sexual scripts and adaptive coping strategies. The literature describes interventions based on directed masturbation and work on changing sexual scripts, which may lead to substantial improvements in orgasmic functioning and overall sexual satisfaction, for example by increasing body awareness and knowledge of preferred forms of stimulation (7). In this context, sex toy use can be viewed as a self-initiated, self-help strategy aimed at facilitating orgasm, enhancing its intensity and improving the subjective quality of sexual life.

Early adulthood is a period of intense sexual exploration, during which identity, patterns of intimacy and erotic preferences are shaped. Population-based studies show that individuals in this age group are more likely to display both greater openness to new sexual experiences and a tendency to engage in behaviors considered risky, such as frequent partner change, unprotected sex or experimenting with diverse forms of sexual activity (8). These phenomena are described as part of typical sexual development in adolescence and early adulthood, although they may be associated with increased health and psychosocial risks (9). Incorporating sex toys into the sexual lives of young women may therefore be interpreted as one expression of this exploratory process, which can coexist both with behaviors that support psychosexual wellbeing and with a broader profile of sexually risky behaviors, assessed, among others, with tools such as the Sexual Risk Survey.

Growing attention is also being paid to hygiene and safety issues. Users report a need for reliable medical information, while healthcare professionals point to gaps in health counseling related to sex toys (10, 11). At the same time, research consistently shows that orgasm frequency is not uniform across groups: women, especially heterosexual women, experience regular orgasms less often than men, which may be linked to dominant patterns of stimulation during intercourse (12). In this context, the role of sex toys, which allow for more varied stimulation, becomes particularly salient (13).

Recent population-based and clinical studies further demonstrate that using sex toys in partnered sex is associated with greater orgasm intensity and higher sexual satisfaction, without being linked to mental health problems (14). Importantly, this phenomenon is also observed among older women, for whom masturbation with sex toys is common and may co-occur with better indices of sexual comfort in the peri- and postmenopausal period (15).

Existing research on sex toy use has focused primarily on populations from Anglophone countries and Western Europe, whereas data from Central and Eastern Europe—including Poland—remain limited. The Polish socio-cultural landscape, characterized by a complex interplay of traditional religious values and evolving modern social norms, offers a unique backdrop for examining female sexuality. Cultural context, levels of religiosity, the quality of sex education and social norms regarding women's sexuality may substantially affect both actual behaviors and the willingness to disclose them in scientific studies. Despite increasing sexual openness among young adults, comprehensive sex education remains limited in the national curriculum, often leaving individuals to rely on self-initiated exploration and technological aids to navigate their sexual well-being. Polish research on sexuality and reproductive health knowledge indicates persistent deficiencies in sex education and diverse patterns of sexual behavior among young women, which further underscores the need for studies that take the local socio-cultural context into account (16, 17). In this light, analyzing sex toy use in a sample of Polish female students not only helps to fill a gap in the international literature but also provides insight into how erotic accessories are embedded within specific cultural and normative conditions.

Despite the growing body of research, detailed data on the prevalence of sex-toy–assisted orgasm and its direct links with different dimensions of sexual quality of life remain limited. The aim of this article is to address this gap by determining how often sex-toy–assisted orgasm occurs in a population of young adult women, examining sex toy use in relation to sexual satisfaction and sexual dysfunction, and exploring factors that may modify or explain these associations. We hypothesize that: (1) frequent partnered sex toy use is associated with higher sexual satisfaction and global sexual quality of life (enhancement function); and (2) a high perceived necessity of such aids correlates with domain-specific challenges, such as lubrication difficulties or elevated orgasmic thresholds, potentially serving as a functional adaptation (compensatory function) rather than an indicator of global sexual dysfunction.

## 2 Material and methods

### 2.1 Study context and objectives

The current research was conducted as a specialized analysis within the broader “PolSex2024” initiative, which explores the sexuality of university students in Poland. While the initial report from this cohort focused on relation between sexual quality of life and symptoms of sexual dysfunctions, the current paper specifically investigates the intersection of erotic accessory usage, orgasmic function, and sexual risk behaviors (18).

Data were gathered through an anonymous, cross-sectional online survey (Google Forms) between March and November 2024. Strict participant anonymity was maintained to encourage honest disclosure regarding sensitive sexual practices.

### 2.2 Participants and recruitment

A multi-stage, non-probability convenience sampling method was employed to reach female students across randomly selected 20 major Polish academic centers. Recruitment involved digital outreach to social media coordinators of student-focused groups (Facebook), where the survey link was distributed following administrative consent. Eligibility was limited to individuals who (1) identified as female at birth, (2) were currently enrolled in a Polish university, (3) were aged 18 or older, and (4) had a history of sexual initiation. In this study, sexual initiation was defined broadly as the first instance of engaging in partnered sexual activity, encompassing a spectrum of practices including petting, oral sex, and penetrative intercourse.

### 2.2 Ethics

The study involved human subjects and was conducted in accordance with the Declaration of Helsinki. According to the Bioethics Committee of the Medical University of Silesia (Decision No. BNW/NWN/0052/KB/213/23), formal ethical approval was waived as the study was non-interventional, anonymous, and voluntary in nature.

### 2.3 Measures

The study utilized a comprehensive set of research instruments comprising four distinct sections:

#### 2.3.1 The female sexual function index (FSFI)

To quantify sexual health, we utilized the FSFI, a 19-item multidimensional scale designed to evaluate functioning over the preceding month. The instrument assesses six core dimensions: arousal, desire, lubrication, orgasm, pain and satisfaction. Weighted domain scores were aggregated into a composite index ranging from 2.0 to 36.0, with higher results indicating enhanced sexual wellbeing. Following established psychometric standards, a cumulative score of 26.55 or less was defined as the clinical threshold to categorize participants at an increased risk of sexual dysfunction. (19, 20).

#### 2.3.2 Sexual risk survey (SRS)

The study utilized the Sexual Risk Survey (SRS), developed by Turchik and Garske (21), using the Polish language translation by Dr. Aleksandra Barabasz-Gembczyk. This 23-item instrument assesses predispositions and actual risk-taking sexual behaviors in the young adult population. The analysis allowed for the calculation of five factors defining risky sexual behaviors occurring in the last 6 months: sexual behaviors with casual partners (F1), risky sexual intercourse (F2), impulsive sexual behaviors (F3), intentions to engage in risky behaviors (F4), and risky anal intercourse (F5). Both a total score and separate scores for each domain are calculated. The tool is characterized by satisfactory psychometric properties. (21, 22).

#### 2.3.3 Original sexual behavior and sex toy questionnaire

The third module consisted of an original, multi-dimensional instrument designed to capture a detailed retrospective sexual history and current behavioral patterns. This section was structured into three thematic blocks:

**General sexual history and practices:** Participants provided data on their current relationship status and the total number of sexual partners, both over their lifetime and within the last 12 months. We also recorded the frequency of sexual intercourse and the chronological age of initiation for a spectrum of sexual activities, including masturbation, petting, and oral, vaginal, and anal intercourse. Furthermore, general sexual wellbeing was assessed through the frequency of lubricant use and a 5-point Likert-scale item measuring global sexual satisfaction.

**Sex toy usage patterns:** A significant portion of the tool focused on the integration of erotic accessories into intimacy. The frequency of sex toy use was assessed independently for solo activities (masturbation) and partnered sexual encounters. Each item utilized a descriptive 5-point Likert scale, ranging from 1 (“Never”) to 5 (“Almost always”). A pivotal variable was the “perceived necessity of a sex toy for orgasm,” which evaluated the degree of subjective reliance on electronic aids to reach climax, also measured on a 5-point scale (ranging from 1 = “Never” to 5 = “Always”).

**Clinical screening indicators:** Participants were queried using dichotomous items (Yes/No) regarding the presence of any self-perceived symptoms of sexual dysfunction experienced within the last year. Additionally, respondents provided observations on perceived sexual difficulties in their partners, such as erectile dysfunction or premature ejaculation, as noted during intimate encounters.

#### 2.3.4 Demographics

The final section gathered basic sociodemographic data, including age, field of study, place of origin and current residence (stratified by population size). Participants were further asked about their living arrangements, parental status, gender assigned at birth and sexual orientation.

### 2.4 Statistics

Data management and computational procedures were executed using Statistica 13.3 software (StatSoft, Kraków, Poland). To ensure the robustness of the findings, a *post-hoc* power calculation was performed, confirming that the study's sample size (*N* = 199) was sufficient to observe small-to-moderate effect sizes (*r* > 0.20 and *f*^2^ > 0.06) with a statistical power exceeding 80% at a significance level of α= 0.05.

The distribution of continuous variables was evaluated via the Shapiro-Wilk test, which indicated a non-normal distribution for the majority of the analyzed parameters. Consequently, non-parametric inferential statistics were applied throughout the study. Numerical data were reported as means ± standard deviations (SD) or medians, while categorical parameters were expressed as absolute frequencies and percentages.

Comparative analyses between independent subgroups were conducted using the Mann-Whitney U test or Kruskal-Wallis ANOVA for multiple groups. Associations between continuous variables were quantified through Spearman's rank correlation coefficient. For the assessment of qualitative variables, the Chi-square test was utilized. To maintain a rigorous threshold for significance across multiple tests and mitigate Type I error, the Benjamini-Hochberg procedure was applied to control the false discovery rate (FDR).

Independent determinants of the “perceived necessity of a sex toy for orgasm” and FSFI domains were identified via a multivariable linear regression model using backward elimination of the variables. Potential predictors with *P* < 0.15 in univariate screening were entered into the model. Prior to final model construction, collinearity was evaluated using the Variance Inflation Factor (VIF), with a retention threshold of VIF < 5 to ensure model stability. For all statistical procedures, a *P*-value of < 0.05 was considered the threshold for statistical significance.

## 3 Results

### 3.1 Sample profile

After excluding 12 incomplete responses and participants not meeting the criteria, the final analysis included 199 female students, with a mean age of 26.2 ± 6.2 years. While a significant portion of the sample (nearly half) originated from smaller municipalities with fewer than 50,000 residents, the current residence patterns indicate a strong shift toward large academic centers; approximately seven out of ten respondents now live in cities exceeding 100,000 inhabitants. Residential independence was high, with 70% living away from their family home, including 33.7% who cohabit with a partner. Parenthood was reported by 12.6% of the group. Regarding personal values, approximately 22% of participants identified as practicing Roman Catholics. The majority of the sample (78.4%) identified as heterosexual, while the remaining participants reported bisexual (16.6%), homosexual (2.0%), or other sexual orientations. The demographic profile of the study sample is presented in Table 1.

**Table 1:** Demographic profile of the sample.

Variable		%
**N**		199
**Age [mean** **±** **SD]**		26.2 ± 6.2
**Field of study**	Artistic/Agriculture/Natural sciences/Sports	9%
	STEM & Technical	18.6%
	Humanities/Law/Arts	32.6%
	Medical sciences	36.7%
	Other	12.1%
**The town of origin:**	village	21.6%
	< 50.000 residents	27.2%
	50.000–100.000 residents	14.6%
	100.000–200.000 residents	18.6%
	>200.000 residents	18.1%
**The place of current residence:**	village	9.5%
	< 50.000 residents	12.5%
	50.000–100.000 residents	9.5%
	100.000–200.000 residents	26.6%
	>200.000 residents	41.7%
**Form of residence:**	With a partner	33.7%
	Shared apartment/Dorm:	23.1%
	Family home	29.6%
	Alone	13.6%
**Sexual orientation**	Heterosexual	78.4%
	Bisexual	16.6%
	Homosexual	2.0%
	Other	3.0%

### 3.2 Relationship status

Monogamous patterns were dominant in the group, with 81.9% of students maintaining a single regular sexual partner. Married participants, representing 10.1% of the sample, exhibited distinct patterns in their intimate lives. Specifically, the degree of relationship formalization significantly influenced the shared use of erotic accessories (Kruskal-Wallis test; *H* = 13.5; *P* = 0.001). *Post-hoc* comparisons revealed that married women integrated sex toys into partnered activity more frequently than those in non-marital steady relationships or those without a permanent partner (mean: 3.0 ± 1.5 vs 1.8 ± 1.1; *Z* = 3.1; *P* = 0.005 and mean: 3.0 ± 1.5 vs 1.8 ± 1.1; *post-hoc*; *Z* = 3.2; *P* = 0.004).

### 3.3 Spectrum of sexual activities

Weekly sexual intercourse was the norm for 60% of the respondents, with one-fifth reporting high-frequency activity (more than once every 2 days). While vaginal and oral sex, as well as petting and masturbation, were near-universal experiences, anal intercourse was reported by a smaller subset of 44.2%. Initiation ages followed a chronological sequence: participants typically began petting, oral, and vaginal sex at approximately 18 years of age, whereas anal initiation occurred significantly later, with an average age of 21 (SD = 4.7). The median lifetime partner count was 2 (IQR: 1–4), though short-term partner stability was high, with most reporting only one partner within the preceding year. Table 2 presents sexual behavior patterns of the Study Group.

**Table 2:** Sexual behavior patterns of the sample.

Variable			%
**N**			199
**Having regular sexual partner**	Yes (one)		81.9%
	Yes (many)		1.5%
	No		16.6%
**Being in a regular relationship:**	Yes, Married		10.1%
	Yes, Partner		45.2%
	No		44.7%
**Frequency of sexual intercourse:**	< 1x/month		16.8%
	1–2x/month		22.5%
	1–2x/week		37.7%
	3–4x/week		14.1%
	> 4x/week		8.9%
**Have you engaged in the following sexual practices?**	Masturbation	Yes	92.0%
		No	8.0%
	Petting	Yes	96.0%
		No	4.0%
	Oral sex	Yes	91.0%
		No	9.0%
	Vaginal sex	Yes	86.9%
		No	13.1%
	Anal sex	Yes	44.2%
		No	55.8%
**Average age of initiation of sexual practice [years]**	Masturbation		14.5 ± 3.7
	Petting		17.5 ± 2.3
	Oral sex		18.1 ± 2.2
	Vaginal sex		18.1 ± 2.5
	Anal sex		21.0 ± 4.7
**Number of sexual partners so far** ^*^			[1]−2–[4]
**Number of sexual partners in the last 12 months** ^*^			[1]−1–[1]

^*^[1 quartile]–median–[3 quartile].

### 3.4 Characteristics of sex toy usage

The section of the questionnaire regarding sex toys comprised three items: 1) frequency of solo toy use, 2) frequency of use with a partner, and 3) perceived necessity of a sex toy to achieve orgasm. Participants answered each item using a Likert scale anchored with descriptive labels to ensure response validity, as presented in Table 3.

**Table 3:** Sex toy usage in the study sample.

Variable		%
**Frequency of sex toy use during masturbation:** ^*^	1–Never	48.2
	2–Occasionally	11.1
	3–Less than once a week	14.1
	4–Once or more a week	7
	5–Almost always	19.6
**Frequency of sex toy use with a partner:** ^*^	1–Never	55.3
	2–Occasionally	16.1
	3–Less than once a week	16.1
	4–Once or more a week	8.5
	5–Almost always	4
**Perceived necessity of a sex toy for orgasm:** ^*^	1–Never	72.4
	2–Occasionally	14.6
	3–Sometimes	5
	4–Often	3.5
	5–Always	4.5

^*^-Likert Scale 1–5 and descriptions.

#### 3.4.1 General characteristics of sex toy usage

52% of respondents confirmed using sex toys during masturbation, with 20% doing so during every such activity. The study demonstrated that nearly 30% of women use sex toys with a partner at least once a week, while 4% use them during every partnered sexual activity. Complete dependence on a sex toy to achieve orgasm was reported by 4.5% of respondents. Conversely, nearly 30% indicated that a sex toy plays a significant role in their experience of orgasm.

#### 3.4.2 General correlations regarding sex toy usage

A very strong positive correlation was found between the frequency of solo sex toy use during masturbation and the frequency of use with a partner (Spearman's rank correlation; *r* = 0.60; *P* < 0.0001). More frequent use of sex toys (both during masturbation and with a partner) correlated with a higher perceived necessity of a sex toy to achieve orgasm (*r* = 0.50 and *r* = 0.47, respectively; *P* < 0.0001).

#### 3.4.3 Association between the necessity of a sex toy for orgasm and sexual function

A robust negative correlation was demonstrated between the declared necessity of a sex toy and the FSFI orgasm domain (Spearman's rank correlation; *r*=-0.23; *P* < 0.001), which remained significant after FDR correction. Similarly, the perceived necessity of sex toys for orgasm correlated significantly with a lower total FSFI score (*r*=-0.18; *P*=0.01). Although a weak negative trend was observed between the necessity of a sex toy for orgasm and lifetime sexual satisfaction measured on a 5-point Likert scale (unadjusted *P*=0.03), this relationship did not retain statistical significance after applying the FDR correction. Similarly, while the initial analysis suggested a link with the FSFI arousal domain (unadjusted *P*=0.02), this association was not significant after adjustment. No association was found with the desire domain.

#### 3.4.4 Intercourse frequency and number of partners vs. sex toys

An increase in the number of lifetime sexual partners correlated with more frequent use of sex toys both during masturbation and with a partner (Spearman's rank correlation; *r* = 0.34 and *r* = 0.25, respectively; *P* < 0.0001). However, this observation did not apply to the number of partners in the last 12 months. More frequent intercourse correlated with more frequent use of sex toys with a partner (*r* = 0.28; *P* < 0.0001). No relationship was found between the frequency of intercourse and the necessity of a sex toy to achieve orgasm.

#### 3.4.5 Sex toys and lubrication

A higher necessity of sex toys to achieve orgasm correlated with poorer lubrication scores in the FSFI lubrication domain (Spearman's rank correlation; *r* = −0.23 *P* = 0.01) and with more frequent use of lubricants measured on a 5-point Likert scale (*r* = 0.29 *P* < 0.0001). Greater frequency of sex toy use was associated with more frequent lubricant use, both in solo and partnered contexts (Spearman's rank correlation; *r* = 0.34 oraz *r* = 0.25 *P* < 0.0001), but did not correlate with the FSFI lubrication domain.

#### 3.4.6 Age of sexual initiation and sex toys

An association was observed between earlier sexual initiation (petting, oral, and vaginal sex) and more frequent use of sex toys, both during masturbation and with a partner (Spearman's rank correlation; *r* ranging from−0.17 to−0.26; *P* < 0.001). No such dependence was observed regarding the age of first masturbation. Similarly, no relationship was found between the age of sexual initiation and the necessity of using a sex toy to achieve orgasm. Older respondents used sex toys with a partner more frequently (*r* = 0.28; *P* < 0.0001); however, no relationship was observed between age and the frequency of using sex toys for masturbation. Likewise, no link was shown between age and the necessity of a sex toy for orgasm.

#### 3.4.7 Frequency of toy use and sexual quality of life

Higher frequency of sex toy use with a partner was associated with greater sexual satisfaction in the FSFI satisfaction domain (*r* = 0.22; *P* = 0.001). Similarly, more frequent use of sex toys with a partner correlated with a better total FSFI score (r = 0.19; *P* = 0.006). Regarding specific FSFI domains, initial univariate analyses suggested potential weak correlations between toy use with a partner and domains of desire (*P* = 0.03), orgasm (*P* = 0.03), and pain (*P* = 0.03); however, these associations did not survive FDR correction for multiple comparisons. Furthermore, although unadjusted analysis indicated a link between the FSFI desire/arousal domains and toy use during masturbation (*P* = 0.01 and *P* = 0.04), these relationships were considered non-significant or marginal after FDR adjustment.

#### 3.4.8 Sex toys and sexual dysfunction symptoms

A comparative analysis between groups with clinically significant dysfunction (FSFI ≤ 26.55) and those without dysfunction revealed no statistically significant difference in the frequency of sex toy use with a partner after applying FDR correction (unadjusted *P* = 0.039; considered non-significant). However, for masturbation, the difference remained marginal/significant (Mann-Whitney U test; Z = 2.44; *P* = 0.015). No difference was found between groups regarding the necessity of a sex toy to achieve orgasm. Analysis of specific sexual dysfunction symptoms showed that patients reporting problems with genital tract lubrication during intercourse showed a trend toward declaring the necessity of using a sex toy to achieve orgasm (unadjusted *P* = 0.03; non-significant after FDR). Similarly, for respondents reporting premature ejaculation in their partner, the dependence on sex toys for orgasm did not reach statistical significance after correction (unadjusted *P* = 0.02).

#### 3.4.10 Factors affecting the ‘perceived necessity of a sex toy for orgasm'

To identify factors independently associated with the ‘perceived necessity of a sex toy for orgasm', a multivariate linear regression model was constructed using variables that reached significance in the univariate screening. The final model identified four independent predictors that entered the regression equation (Table 4). According to the standard partial regression coefficients, the independent factors affecting the perceived necessity of a sex toy for orgasm were:

1) frequency of sex toy use during masturbation (positive predictor).
2) frequency of sex toy use with a partner (positive predictor).
3) FSFI orgasm domain (negative predictor).
4) presence of symptoms of sexual dysfunction (positive predictor).

**Table 4:** Univariate and multivariate linear regression analysis identifying predictors of ‘Perceived necessity of a sex toy for orgasm'.

Variables	Univariate analysis		Multivariate analysis			
	*r*	*P*	Coefficient	SE	T	*P*
***Linear regression results for ‘Perceived necessity of a sex toy for orgasm'***						
Frequency of sex toy use during masturbation	0.51	< 0.0001	0.22	0.05	4.63	0.000007
Frequency of sex toy use with a partner	0.47	< 0.0001	0.25	0.06	3.94	0.0001
FSFI orgasm domain (no 4)	−0.23	0.0009	−0.13	0.04	−3.08	0.002
Presence of symptoms of sexual dysfunction	0.20	0.004	0.34	0.15	2.27	0.024
Frequency of lubricant use during intercourse	0.29	< 0.0001				NS
Number of symptoms of sexual dysfunction	0.17	0.01				NS
Presence of premature ejaculation in the partner ^*^	0.17	0.015				NS
Problem with lubrication of the genital tract during intercourse^*^	0.15	0.03				NS
Satisfaction with sexual life (lifetime)	−0.15	0.03				NS
FSFI score	−0.18	0.01				NS
FSFI arrousal domain (no 2)	−0.18	0.02				NS
FSFI lubrication domain (no 3)	−0.23	0.001				NS

^*^(observed in partner by respondent in last 12 months).

#### 3.4.11 Impact of ‘Perceived Necessity of a Sex Toy for Orgasm' on Specific FSFI Domains

A series of multivariate linear regression models were conducted to determine the independent influence of “perceived necessity of a sex toy for orgasm” on various dimensions of sexual functioning as expressed by individual FSFI domains.

The multivariate analysis revealed that the ‘perceived necessity of a sex toy for orgasm' is a significant independent negative predictor for three specific FSFI domains: arousal, lubrication and orgasm (Table 5).

**Table 5:** Univariate and multivariate linear regression analysis identifying predictors of Female Sexual Functioning Index (FSFI) domains.

Variables	Univariate analysis		Multivariate analysis			
	***r***	***P***	**Coefficient**	**SE**	**T**	***P***
*Linear regression results for FSFI arrousal domain (no 2)*						
Frequency of sexual intercourse	0.33	< 0.00001	0.32	0.08	4.09	0.00006
Satisfaction with sexual life (last 12 months)	0.40	< 0.00001	0.25	0.09	2.93	0.004
Frequency of sex toy use during masturbation	0.14	0.045	0.19	0.06	3.03	0.003
Number of symptoms of sexual dysfunction	−0.35	< 0.00001	−0.12	0.06	−2.01	0.046
Perceived necessity of a sex toy for orgasm	−0.16	0.024	−0.21	0.09	−2.22	0.03
Number of sexual partners (last 12 months)	0.19	0.007				NS
Being in a regular relationship	0.17	0.019				NS
*Linear regression results for FSFI lubrication domain (no 3)*						
Frequency of sexual intercourse	0.25	0.00045	0.42	0.08	5.56	< 0.00001
Perceived necessity of a sex toy for orgasm	−0.23	0.001	−0.24	0.09	−2.73	0.0068
Number of sexual partners in last 12 months	0.17	0.016	0.24	0.12	2.01	0.046
Satisfaction with sexual life (last 12 months)	0.23	0.001				NS
Number of symptoms of sexual dysfunction	−0.28	0.00005				NS
Frequency of lubricant use during intercourse	−0.25	0.0004				NS
*Linear regression results for FSFI orgasm domain (no 4)*						
Satisfaction with sexual life (last 12 months)	0.46	< 0.00001	0.43	0.10	4.49	0.00001
Frequency of sexual intercourse	0.36	< 0.00001	0.27	0.09	2.97	0.003
Perceived necessity of a sex toy for orgasm	−0.23	0.0009	−0.26	0.09	−2.83	0.005
Age	0.17	0.015	0.04	0.02	2.57	0.01
Being in a regular relationship	0.18	0.009				NS
Frequency of sex toy use with a partner	0.15	0.035				NS
Number of symptoms of sexual dysfunction in partner	−0.14	0.048				NS
*Linear regression results for FSFI score*						
Frequency of sexual intercourse	0.51	< 0.00001	1.9	0.31	6.13	< 0.00001
Number of symptoms of sexual dysfunction	−0.49	< 0.00001	−1.0	0.23	−4.42	0.00002
Satisfaction with sexual life (last 12 months)	0.54	< 0.00001	1.3	0.34	3.79	0.0002
Number of sexual partners in last 12 months	0.20	0.004	0.9	0.42	2.04	0.04
Being in a regular relationship	0.24	0.0006				NS
Frequency of sex toy use with a partner	0.19	0.006				NS
Perceived necessity of a sex toy for orgasm	−0.18	0.01				NS

##### 3.4.11.1 Factors affecting the FSFI arousal domain

Beyond the perceived necessity of a sex toy for orgasm, the multivariate model identified four other independent predictors for the FSFI arousal domain: 1) frequency of sexual intercourse (positive predictor) 2) sexual satisfaction (positive predictor) 3) frequency of toy use during masturbation (positive predictor) 4) number of dysfunction symptoms (negative predictor).

##### 3.4.11.2 Factors affecting the FSFI lubrication domain

For lubrication domain, beyond the perceived necessity of a sex toy for orgasm, independent predictors included: 1) frequency of sexual intercourse (positive predictor) 2) number of sexual partners in the last 12 months (positive predictor).

##### 3.4.11.3 Factors affecting the FSFI orgasm domain

In the orgasm domain regression model, beyond the perceived necessity of a sex toy for orgasm, as independent predictors were indentified: 1) satisfaction with sexual life in last 12 months (positive predictor) 2) frequency of sexual intercourse (positive predictor) 3) age (positive predictor).

##### 3.4.11.4 Factors affecting the FSFI total score

Notably, while the perceived necessity of a sex toy significantly influenced individual domains of FSFI, it did not emerge as an independent predictor of the total FSFI score.

The total FSFI score was instead independently predicted by:

1) frequency of sexual intercourse (positive predictor).
2) satisfaction with sexual life in last 12 months (positive predictor).
3) number of sexual partners in the last 12 months (positive predictor).
4) number of dysfunction symptoms (negative predictor).

### 3.5 Sexual risk survey (SRS)

#### 3.5.1 General characteristics of risky behaviors

Women who engaged in risky sexual behaviors significantly more often reported a higher quality of sexual life according to the FSFI (Spearman's rank correlation; *r* = 0.18; *P* = 0.01). It was observed that the SRS score correlated most strongly with the FSFI desire domain (*r* = 0.26; *P* < 0.001). Women who reported dyspareunia less frequently engaged in risky sexual behaviors more often (*r* = 0.20; *P* = 0.005). A higher SRS score significantly correlated with a greater number of sexual partners in a lifetime (*r* = 0.69; *P* < 0.001) and in the last 12 months (*r* = 0.38; *P* < 0.001), as well as with a higher frequency of intercourse (*r* = 0.27; *P* < 0.001). Older respondents had significantly higher SRS scores (*r* = 0.27; *P* < 0.001). Individuals who more strongly guided their lives by Catholic religious principles engaged in risky sexual behaviors significantly less often (*r* = −0.34; *P* = 0.003).

#### 3.5.2 . Age of sexual initiation and SRS

Respondents who initiated sexual activity earlier showed a higher tendency to engage in risky sexual behaviors. This correlated with the age of first vaginal sex (Spearman's rank correlation; *r* = −0.45; *P* < 0.001), petting (*r* = −0.39; *P* < 0.001), oral sex (*r* = −0.35; *P* < 0.001), and anal sex (*r* = −0.21; *P* = 0.008). No relationship was demonstrated between the age of first masturbation and engagement in risky sexual behaviors.

#### 3.5.3 Sex toys and risky sexual behaviors

Table 6 presents the relationships between sex toy use and SRS scores.

**Table 6:** Correlation between SRS score and use of sex toy.

SRS domain	Frequency of sex toy use during masturbation	Frequency of sex toy use with a partner	Perceived necessity of a sex toy for orgasm
**F1** (sexual behaviors with casual partners)	*r* = 0.31 *P < 0.0001*	*r* = 0.22 *P = 0.002*	NS
**F2** (risky sexual intercourse)	*r* = 0.23 *P = 0.001*	*r* = 0.20 *P = 0.004*	NS
**F3** (impulsive sexual behaviors)	*r* = 0.17 *P = 0.02*	NS	NS
**F4** (intentions to engage in risky behaviors)	*r* = 0.17 *P = 0.02*	NS	NS
**F5** (risky anal intercourse)	*r* = 0.27 *P < 0.0001*	*r* = 0.31 *P < 0.0001*	NS
**SRS-score**	*r* = 0.32 *P < 0.0001*	*r* = 0.25 *P = 0.0004*	NS

[Spearman's Rank Correlation: r = coefficient; P= P-Value].

A significant relationship was observed between a higher frequency of sex toy use during masturbation and a greater propensity for risky sexual behaviors, observed in both the total SRS score and across all SRS domains. Similarly, a higher frequency of sex toy use with a partner was associated with a higher total SRS score and higher scores in the domains of casual partner behaviors (F1), risky sexual intercourse (F2), and risky anal intercourse (F5). The above relationships were not observed between risky sexual behaviors and the necessity of a sex toy to achieve orgasm.

## 4 Discussion

This study examined the prevalence of sex toy use among young adult women in Poland and investigated its associations with sexual functioning, satisfaction, and risk behaviors. Our findings highlight a dual role for erotic accessories: as tools for intimacy enhancement in partnered relationships and as functional, compensatory aids used to address specific physiological thresholds.

A central focus of this analysis was the “perceived necessity” of sex toys for reaching orgasm, a concept that we have reframed as “functional necessity” to avoid stigmatizing common sexual practices. While complete reliance on a toy was reported by only 4.5% of the sample, nearly 30% indicated that these devices play a significant role in their experience of climax. Crucially, our multivariate regression models revealed that while “perceived necessity” was a significant independent negative predictor for the arousal, lubrication and orgasm domains, it did not emerge as an independent predictor of the total FSFI score. This distinction is vital - it suggests that reliance on a toy often represents a functional adaptation to reach a physiological threshold—such as the need for consistent clitoral stimulation—rather than a global impairment of sexual quality of life (13). However, for a subset of women, this necessity may coexist with an ambivalent psychological burden regarding “natural” responsiveness (23–25).

Epidemiological data from the past decade indicate that sex toy use among women is common and occurs both in solo contexts and within partnered relationships. In our study, half of the participants reported using sex toys regularly during masturbation, while almost one-third used them during sexual activity with a partner. These results align with national surveys from other European countries where “ever use” rates for solo and partnered activity frequently exceed 45% (1). Furthermore, the finding that married women in our cohort used toys more frequently with their partners reflects broader European trends where women in stable relationships report higher integration of erotic accessories (2).

Our results confirm that incorporating sex toys into partnered sex serves as a positive intimacy-enhancing strategy. Respondents who used toys with a partner reported significantly higher sexual satisfaction and better total FSFI scores. This mirrors literature associating partnered toy use with greater orgasmic intensity and higher relational satisfaction without links to psychopathology (14, 26). By expanding the repertoire of shared stimulation, these accessories likely contribute to more favorable evaluations of the sexual relationship.

We observed that a higher number of lifetime sexual partners was associated with more frequent sex toy use in both solo and partnered contexts. In addition, higher coital frequency positively correlated with more frequent inclusion of erotic accessories in partnered sexual activity. These relationships are consistent with global findings showing that populations characterized by higher sexual activity, stronger desire, and more frequent pornography viewing more often report sex toy use, interpreted as an indicator of sexual openness and a drive to enhance pleasure and relational satisfaction (26). In the same study, sex toy use was associated with better sexual quality of life, with a stronger effect in partnered sex than in solo activity (26).

The association between toy use and lubrication in our cohort further supports this compensatory interpretation. We found that a higher perceived necessity of toys independently predicted poorer lubrication scores and more frequent lubricant use. This is consistent with data showing that lubricants are frequently used alongside vibrators to enhance arousal and manage physiological thresholds (27). While clinical interventions utilizing vibratory stimulation have been shown to improve overall sexual functioning scores, our findings suggest that in a non-clinical population, the subjective “necessity” of a toy may specifically signal a functional response to underlying lubrication difficulties (3, 28).

In our comparative analysis, women without clinically relevant sexual dysfunction more often used sex toys during masturbation, a finding that survived FDR correction, yet no significant difference was observed in the frequency of partnered use or in the perceived necessity of the device for orgasm. Similarly, Rullo et al. reported that more frequent inclusion of vibratory stimulation, provided that the device is appropriately selected and adequate education is offered, is associated with improved sexual functioning [13]. This effect was also confirmed in the study by Guess et al., where a vibrator-based intervention led to a significant improvement in FSFI scores (28).

In our sample, more frequent sex toy use—both in autoerotic and partnered activity—was associated with higher SRS scores, indicating a greater tendency toward sexually risky behaviors. This association involved both the total SRS score and specific domains related to casual partners, risky sexual practices, and anal behaviors. Similar trends have been reported previously: in a study on so-called sexual enrichment aids (SEA), women using such accessories more often engaged in diverse sexual practices, not always with protective barriers, potentially increasing exposure to sexually transmitted infections (10). Marrazzo et al. further showed that sharing intravaginal sex toys without protection is associated with a significantly increased risk of bacterial vaginosis, underscoring the importance of appropriate hygiene and barrier methods in infection prevention (29). Taken together, these findings suggest that intensified sex toy use may co-occur with more open but potentially risky sexual behavior patterns, which should be examined further in prospective studies.

Our data also support age as an important factor differentiating patterns of sex toy use. Older respondents more frequently integrated sex toys into partnered sexual activity, whereas no association was found between age and sex toy use in solo contexts. A similar pattern was described in an Indian population, where respondents aged 25–44 years were approximately twice as likely to use sex toys as younger participants aged 18–24 (30). This suggests that with increasing age, individuals may become more inclined to incorporate erotic accessories into partnered sex, possibly reflecting greater sexual experience and openness to shared exploration of stimuli.

To date, no studies have directly examined the relationship between premature ejaculation (PE) in male partners and sex toy use among women. While our univariate analysis initially suggested that women reporting PE in their partners more often indicated a greater dependence on sex toys to achieve orgasm, this association did not reach the strict threshold for statistical significance after applying the FDR correction. Therefore, we cannot conclude a significant direct relationship based on these data. The observed trend may reflect a compensatory function of these devices in the context of shortened intercourse and insufficient stimulation. The literature does, however, include data on vibratory start–stop therapy as a treatment for PE. A randomized clinical trial showed that this form of controlled vibratory stimulation significantly prolonged ejaculation latency and improved control over ejaculation, representing an effective adjunct to behavioral therapy (31, 32). In light of our findings, it can still be hypothesized that vibratory devices may have therapeutic value not only for men with PE but also a supportive role for their partners, enabling more complete sexual satisfaction despite limitations associated with PE. Further studies with larger samples are needed to confirm the potential compensatory role suggested by the initial trend.

The Polish socio-cultural context is fundamental to these findings. Our sample included students from various academic centers, with a significant portion originating from smaller municipalities where conservative or religious values may be more prevalent. In a society where comprehensive sex education remains limited, sex toys may serve as accessible tools for self-initiated sexual exploration and anatomy education. While religious principles were associated with fewer risky behaviors, the overall prevalence of toy use suggests that these accessories are becoming normalized even within traditional demographic segments.

This study has clear implications for public health practice and education. Health promotion programs in universities should move beyond risk-reduction to include the healthy integration of enhancement aids into sexual well-being. Clinicians should adopt a stigma-sensitive, non-pathologizing approach, recognizing that the “necessity” of a toy often represents an adaptive strategy to achieve physiological climax

### 4.1 Limitations

The present study makes a meaningful contribution to understanding sex toy use and sexual functioning in women; however, it should be interpreted in light of several important methodological limitations. First, its cross-sectional design precludes conclusions about causal relationships between sex toy use, orgasm, and sexual quality of life. Second, the data are based on self-report, which carries a risk of cognitive biases, incomplete responses, and the influence of socially desirable responding. Furthermore, the response rate could not be accurately determined because the survey link was distributed through various social media platforms, preventing an estimation of the total recruitment reach.

The sample consisted of female university students in Poland, which limits the generalizability of the findings to the broader population, including individuals outside academic settings and older women. A self-selection effect is also likely: women with greater sexual openness or a stronger interest in sexology may have been more inclined to participate. In addition, some of the study instruments, although previously reviewed by experts, were study-specific and not formally validated, which may affect the precision of measurement for certain variables.

Moreover, we did not examine in detail the specific types of sex toys used, their functions, or the contextual patterns of use, all of which might differentiate the observed associations. In view of these limitations, the findings should be considered exploratory. Nevertheless, they provide an important starting point for prospective research on the relationships between the use of erotic accessories, sexual functioning, and psychosexual wellbeing in women. The question of where the boundary lies between healthy, toy-enhanced sexual pleasure and a problematic reliance on sex toys to attain sexual satisfaction remains unanswered.

## 5 Conclusions

1. Sex toy use is prevalent among young women and constitutes an integral part of contemporary sexual activity.
2. Integrating sex toys into partnered activity is associated with higher sexual satisfaction and improved overall sexual quality of life.
3. The ‘perceived necessity of a sex toy for orgasm' serves as a significant independent negative predictor for specific physiological domains, including arousal, lubrication, and orgasm.
4. Notably, the “perceived necessity” of a sex toy does not independently predict the total FSFI score; this suggests that subjective reliance on these devices represents a functional adaptation to reach physiological thresholds rather than a marker of global sexual impairment.
5. Frequent sex toy use, particularly in solo contexts, acts as an independent positive predictor of sexual arousal scores, supporting its role in healthy sexual exploration and self-knowledge.
6. Higher frequency of sex toy use is independently associated with an increased propensity for sexually risky behaviors across multiple domains, highlighting the urgent need for targeted sexual health education focused on safe exploration, barrier protection, and hygiene.

## Data Availability

The raw data supporting the conclusions of this article will be made available by the authors, without undue reservation.

## References

[1] Döring N Pöschl S. Experiences with diverse sex toys among German heterosexual adults: findings from a national online survey. J Sex Res. (2020) 57:885–96. doi: 10.1080/00224499.2019.1578329 30806076

[2] Hald GM Øverup CS Pavan S. Do sex toys make me satisfied? The use of sex toys and sexual, life, and relationship satisfaction in six European countries. J Sex Res. (2025) 62:735–49. doi: 10.1080/00224499.2024.2304575 38294998

[3] Dubinskaya A Eilber KS Kwon O Rosenblum N Goldman HB Anger JT. The role of vibrators in women's pelvic health: an alluring tool to improve physical, sexual, and mental health. Int Urogynecol J. (2024) 35:1085–92. doi: 10.1007/s00192-024-05775-7 38668760 PMC11150285

[4] Dubinskaya A Horwitz R Scott V Anger J Eilber K. Is it time for doctors to Rx vibrators? A systematic review of pelvic floor outcomes. Sex Med Rev. (2023) 11:15–22. doi: 10.1093/sxmrev/qeac008

[5] Lew-Starowicz Z Czajkowska K. Prevalence of sexual dysfunctions and associated risk factors in Poland. Arch Med Sci. (2019) 18:1031–40. doi: 10.5114/aoms.2019.86794 35832714 PMC9266947

[6] Thomas HN Thurston RC. A biopsychosocial approach to women's sexual function and dysfunction at midlife: a narrative review. Maturitas. (2016) 87:49–60. doi: 10.1016/j.maturitas.2016.02.009 27013288 PMC4808247

[7] Kozakiewicz A. Therapeutic practices for female anorgasmia - a cognitive-behavioral perspective. Psychoterapia. (2024) 208:13–24. doi: 10.12740/PT/191819

[8] Stokłosa I Stokłosa M Porwolik M Gawron A Skrzypulec-Plinta V. Analysis of high-risk sexual behavior among Polish university students. Int J Environ Res Public Health. (2021) 18:3737. doi: 10.3390/ijerph18073737 33918452 PMC8038304

[9] Boislard MA van de Bongardt D Blais M. Sexuality (and lack thereof) in adolescence and early adulthood: a review of the literature. Behav Sci. (2016) 6:8. doi: 10.3390/bs6010008 26999225 PMC4810042

[10] Collar AL Fuentes JE Brakey HR Frietze KM. Sexual enrichment aids: a mixed methods study evaluating use, hygiene, and risk perception among women. J Sex Res. (2022) 59:1153–62. doi: 10.1080/00224499.2021.2015568 34919465 PMC9203601

[11] Collar AL Rishel Brakey H Fuentes JE Frietze KM. Medical counseling on sexual enrichment aids: women's preferences and medical practitioner expertise. Obstet Gynecol. (2022) 140:489–98. doi: 10.1097/AOG.0000000000004892 35926212 PMC9669100

[12] Frederick DA John HKS Garcia JR. Lloyd EA. Differences in orgasm frequency among gay, lesbian, bisexual, and heterosexual men and women in a US national sample. Arch Sex Behav. (2018) 47:273–88. doi: 10.1007/s10508-017-0939-z 28213723

[13] Rullo JE Davenport MT Zhang S Herbenick D. Genital vibration for sexual function and enhancement: best practice recommendations for choosing and safely using a vibrator. Sex Relatsh Ther. (2018) 33:275–85. doi: 10.1080/14681994.2017.1419558 33223961 PMC7678780

[14] Sansone A Mollaioli D Colonnello E Ciocca G Limoncin E Jannini EA. Toys in the bedroom: use of sexual devices in partnered sexual activity is associated with higher female orgasmic intensity, arousal, and sexual satisfaction and is not related to psychopathologies. J Sex Med. (2025) 22:397–403. doi: 10.1093/jsxmed/qdaf004 39916367

[15] Graham CA Ferrall L Lehmiller JJ. Masturbation frequency and experiences among US women aged 40-65 years: comparisons across different stages of the menopause transition. Menopause. (2025) 32:903–12. doi: 10.1097/GME.0000000000002597 40627719 PMC12502948

[16] Warzecha D Szymusik I Pietrzak B Nowakowska D Kiciński M Wielgoś M. Sex education in Poland - a cross-sectional study evaluating over twenty thousand Polish women's knowledge of reproductive health issues and contraceptive methods. BMC Public Health. (2019) 19:689. doi: 10.1186/s12889-019-7046-0 31159803 PMC6547576

[17] Firsiuk K Chachaj W Maciocha A Drogoń J Wdowiak K Gendek K . The sexual behaviors of young Polish women and their knowledge about contraception. Pol J Public Health. (2024) 134:81–8. doi: 10.12923/2083-4829/2024-0018

[18] Stokłosa M Florczyk I Wieckiewicz G Kiersten K Piegza M Pudlo R. Factors influencing the quality of women's sexual life: a study of Polish female students. Healthcare. (2025) 13:3278. doi: 10.3390/healthcare13243278 41464347 PMC12732498

[19] Rosen R Brown C Heiman J Leiblum S Meston C Shabsigh R . The Female Sexual Function Index (FSFI): a multidimensional self-report instrument for the assessment of female sexual function. J Sex Marital Ther. (2000) 26:191–208. doi: 10.1080/009262300278597 10782451

[20] Wiegel M Meston C Rosen R. The Female Sexual Function Index (FSFI): cross-validation and development of clinical cutoff scores. J Sex Marital Ther. (2005) 31:1–20. doi: 10.1080/00926230590475206 15841702

[21] Turchik JA Garske JP. The Sexual Risk Survey: development and initial validation. J Sex Res. (2009) 46:375–88. doi: 10.1080/00224490902747974

[22] Barabasz-Gembczyk A. Analiza impulsywności oraz agresji z uwzglednieniem wybranych aspektów behawioralnych u pacjentek z fenotypem A zespołu policystycznych jajników [dissertation]. Katowice: Slaski Uniwersytet Medyczny w Katowicach. (2023).

[23] Marcus BS. Changes in a woman's sexual experience and expectations following the introduction of electric vibrator assistance. J Sex Med. (2011) 8:3398–406. doi: 10.1111/j.1743-6109.2010.02132.x 21205230

[24] Mayr E. Beyond plug and play: the acquisition and meaning of sex toys. Int J Consum Stud. (2021) 45:1234–45. doi: 10.1111/ijcs.12601

[25] Mayr E. Toy stories: the role of vibrators in domestic intimacies. Cult Health Sex. (2022) 24:1735–49. doi: 10.1177/13634607211000194

[26] Marrazzo JM Thomas KK Agnew K Ringwood K. Prevalence and risks for bacterial vaginosis in women who have sex with women. Sex Transmit Dis. (2010) 37:3359. doi: 10.1097/OLQ.0b013e3181ca3cac 20429087 PMC3291172

[27] Hensel DJ Schick V Herbenick D Dodge B Reece M Sanders SA . Lifetime lubricant use among a nationally representative sample of lesbian- and bisexual-identified women in the United States. J Sex Med. (2015) 12:1257–66. doi: 10.1111/jsm.12873 25974238

[28] Guess MK Connell KA Chudnoff S Ark R Yan X Yamaguchi I . The effects of a genital vibratory stimulation device on sexual function and genital sensation. Female Pelvic Med Reconstr Surg. (2017) 23:256–62. doi: 10.1097/SPV.0000000000000357 27918337

[29] Marrazzo JM Thomas KK Agnew K Ringwood K. Prevalence and risks for bacterial vaginosis in women who have sex with women. Sex Transm Dis. (2010) 37:335–9. doi: 10.1097/OLQ.0b013e3181ca3cac 20429087 PMC3291172

[30] Mhatre P Agrawal A Agrawal P Sharma S Bansal R Singh R. Exploring sex toy use among 18-45-year-old Indian adults: insights from a nationwide online survey. Lancet Reg Health Southeast Asia. (2024) 32:100508. doi: 10.1016/j.lansea.2024.100508 39583949 PMC11585771

[31] Ventus D Gunst A Arver S Öberg K Bohm-Starke N Fugl-Meyer KS. Vibrator-assisted start-stop exercises improve premature ejaculation symptoms: a randomized controlled trial. Arch Sex Behav. (2020) 49:1559–73. doi: 10.1007/s10508-019-01520-0 31741252 PMC7300103

[32] Herbenick D Reece M Sanders S Dodge B Ghassemi A Fortenberry JD. Prevalence and characteristics of vibrator use by women in the United States: results from a nationally representative study. J Sex Med. (2009) 6:1857–66. doi: 10.1111/j.1743-6109.2009.01318.x 19453881

